# Neuroendocrine neoplasms of the breast: a review of literature

**DOI:** 10.1007/s00428-024-03856-y

**Published:** 2024-07-09

**Authors:** Federica Vegni, Ilenia Sara De Stefano, Federica Policardo, Pietro Tralongo, Angela Feraco, Angela Carlino, Giulia Ferraro, Qianqian Zhang, Giulia Scaglione, Nicoletta D’Alessandris, Elena Navarra, Gianfranco Zannoni, Angela Santoro, Antonino Mule, Esther Diana Rossi

**Affiliations:** Division of Anatomic Pathology and Histology-Fondazione, Policlinico Universitario “Agostino Gemelli”-IRCCS, Università Cattolica del Sacro Cuore, Largo Francesco Vito, 1, 00168 Rome, Italy

**Keywords:** Neuroendocrine neoplasms, Histology, Immunohistochemistry, Personalized medicine

## Abstract

Primary neuroendocrine neoplasms (NENs) of the breast are characterized by neuroendocrine architectural and cytological features, which must be supported by immunohistochemical positivity for neuroendocrine markers (such as Chromogranin and Synaptophysin). According to the literature, making a diagnosis of primary neuroendocrine breast cancer always needs to rule out a possible primary neuroendocrine neoplasm from another site. Currently, the latest 2022 version of the WHO of endocrine and neuroendocrine neoplasms has classified breast NENs as well-differentiated neuroendocrine tumours (NETs) and aggressive neuroendocrine carcinomas (NECs), differentiating them from invasive breast cancers of no special type (IBCs-NST). with neuroendocrine features. The current review article describes six cases from our series and a comprehensive review of the literature in the field of NENs of the breast.

## Introduction

Primary neuroendocrine neoplasms (NENs) of the breast are characterized by neuroendocrine architectural and cytological features, which must be supported by immunohistochemistry positivity for neuroendocrine markers (such as Chromogranin and Synaptophysin) [1]. Making a diagnosis of primary neuroendocrine breast cancer needs to rule out metastasis from another anatomical site [2]. The Br-NEN classification has undergone substantial changes over the years and in the various editions of the WHO that have followed (2003, 2012). Currently, according to the latest 2019 classification of the WHO of breast tumours, NENs of the breast are classified as well-differentiated neuroendocrine tumours (NETs), aggressive neuroendocrine carcinomas (NECs) and invasive breast cancers of no special type (IBCs-NST) with neuroendocrine features. A similar diagnostic approach is recommended in the WHO blue book (2022) on endocrine and neuroendocrine tumours, in the chapter on neuroendocrine neoplasms in non-endocrine organs [3]. Because of their rarity (< 1% of breast cancers), the literature is not so extensive, and despite continued advances in research, especially in the molecular biology, to date, there are still no specific guidelines regarding their treatment [3].

The true incidence and clinical features of Br-NENs (breast-NENs) are difficult to define, since neuroendocrine (NE) markers are not routinely used in breast cancer diagnosis and, to date, studies have not established a definitive value for immunohistochemistry (IHC) positivity in order to establish a NE differentiation [2]. Incidence is highly variable, from < 0.1 to 5.4%, probably due to the completely different data collection procedures [4, 5].

Specifically, the third version of the WHO classification, published in 2003, was the first one to classify Br-NENs as a distinct pathological entity. They were divided into three categories: solid NECs, small cell/oat cell carcinomas, and large cell carcinomas [6]. They were distinguished by NE morphologic characteristics comparable to those of GI/lung NETs and expression of NE markers in more than 50% of cell populations. The 2012 WHO Working Group included NENs under the category “carcinomas with NE features” (exhibiting morphological features similar to those of NE tumour of GI tract and lung and expressing NE markers, i.e., chromogranin (CgA) and synaptophysin (Syn) [7, 8]. Thus, in the 4th edition (2012), the definition of NE breast cancers was changed to “cancers with NE features” and the threshold of 50% NE markers was removed. Invasive breast carcinomas with NE differentiation, well-differentiated neuroendocrine tumours (WD-NETs), and poorly differentiated small cell carcinomas were recognized as three subtypes of carcinomas with NE features**.** Well-differentiated NETs, despite their morphological similarities with carcinoid tumours of other sites, often lacked the classical nuclear features. NECs exhibited common features of small cell carcinoma (SmCC), rather than large cell NEC (LCNEC). Moreover, invasive BCs with NE differentiation exhibited distinct subtypes, including MC of type B and SPC, as well as IBCs-NST. Additionally, lobular invasive carcinomas with NE differentiation were observed, though this occurred less commonly.

In the most recent WHO classification (5th ed. 2019)), the term NEN is used for all neoplasms with predominately NE differentiation, which is separated into well- and poorly differentiated neoplasms based on the presence of NE histological /immunohistochemical characteristics in more than 90% of the neoplasm. A well-differentiated NEN corresponds to NET, whereas a poorly differentiated NEN corresponds to NEC. Both categories have low/intermediate or high Nottingham histologic grade NE morphology, as well as extensive NE markers expression [5]. In this way, according to the Nottingham grading system, Br-NENs are graded as well-differentiated tumour (G1), intermediate differentiated tumour (G2), or poorly differentiated (G3) carcinoma. Special-type breast carcinomas (BCs) expressing NE markers, such as SPCs and MCs, were removed from the NEN category.

## Method

Two authors independently conducted the online literature search via PubMed, within the last 10 years. This search started on June 1, 2013, and was continued every day before the last search on July 31, 2023. The terms and free text words were combined using Boolean operators (AND, OR). The following search string was used and inserted into the search bars of the previously reported databases: “breast” AND (“neuroendocrine carcinoma” OR “neuroendocrine tumours” OR “neuroendocrine differentiation”). We considered only studies written and published in English. Abstracts, duplicates, incorrectly entered population, and publications without complete data were excluded. The last search was conducted on 07/31/2023, reporting a total of 7152 records on the PubMed database. These records were screened by title and abstract only, and a total number of 2215 articles passed the first screening process. The resulting records were subjected to full-text reading to meet the exclusion criteria. After this last step, a total of 109 articles met the criteria and were included in the quality and summary. To supplement the literature, we report 6 case reports from the Agostino Gemelli IRCCS University Policlinic Foundation.

## Case reports

Cases are all summarized in Table 1. Herein is the detailed description for each of them.

**Table 1:** The table details the cases analyzed in the manuscript

Case n	Age	Diameter (mm)	Localization	Presence of in situ carcinoma	ER/PR/Her2	Ki-67	GATA3	Other immmunohistochemical markers	Histological diagnosis
1*	54	7	UOQ-LB	LIN 1/2	-/-/0	3%	-	/	Metastasis from thyroid medullary carcinoma
		4	UOQ-RB	LIN2	10%/10%/0	5%	-	CrA + Syn +	Metastasis from thyroid medullary carcinoma
2	67	/	UOQ-LB	no	-/-/0	90%	-	AE1/AE3 + CrA + Syn + CK20 + CK7-HMWCK TIF-1-CDX2-p63-p40-Mammoglobin-	Metastasis from Merkel cell carcinoma of the skin
3	46	23	UOQ-LB	no	95%/90%/0	80%	+	SSTR2A +	PD-NEC from the breast
4	40	22	IOQ-LB	DCIS	100%/60%/2 + (NA)	35%	+	CrA + Syn +	IC-N G3 from the breast
5	36	95	UOQ-LB	DCIS-N	-/-/0	70%	+	CrA + Syn + CK7 + TTF-1	PD-NEC from the breast
6	47	3	IOQ-LB	no	95%/95%/2 + (NA)	30%	+	CrA + Syn +	IC-N G2 from the breast

*LB*; left breast, *RB*; right breast, *UOQ*; upper quadrant, *IOQ*; inferior quadrant

### Metastases

#### Case 1

**Fig. 1:**
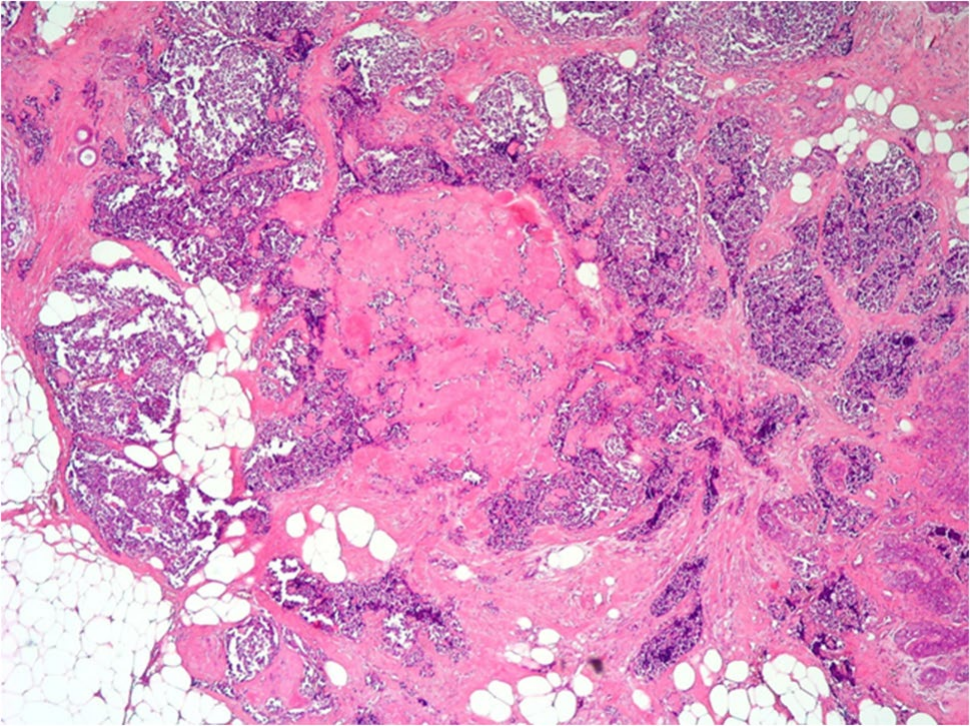
Multiple foci of carcinoma with small cells and neuroendocrine differentiation in breast tissue with numerous foci of LIN1-2 (H&E 200X)

**Fig. 2:**
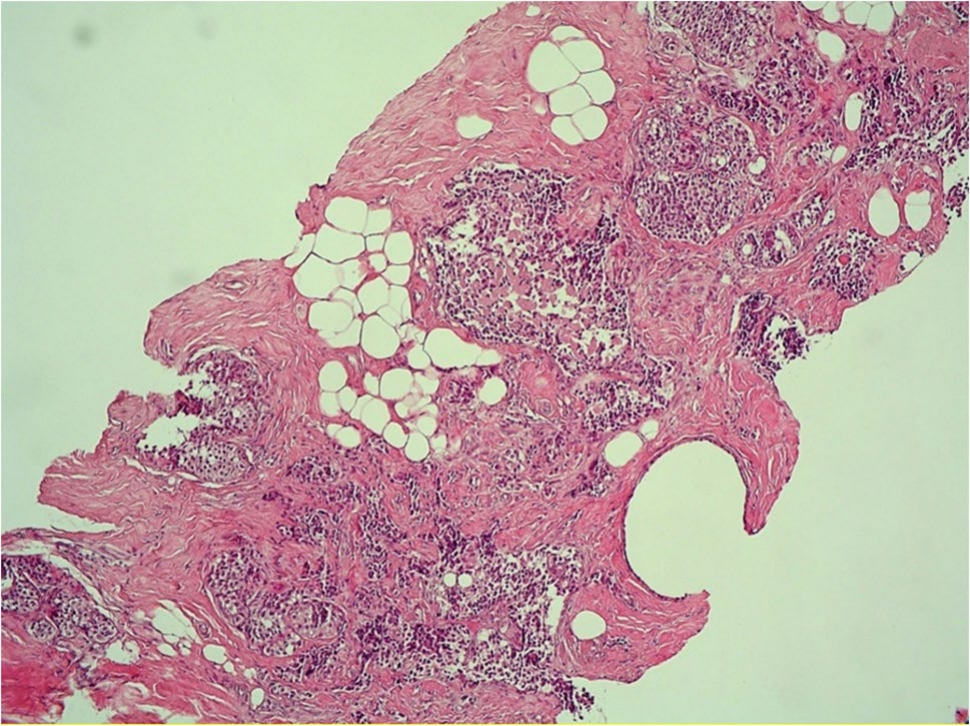
Details of invasive carcinoma with some foci of LIN2 (H&E 400X)

**Fig. 3:**
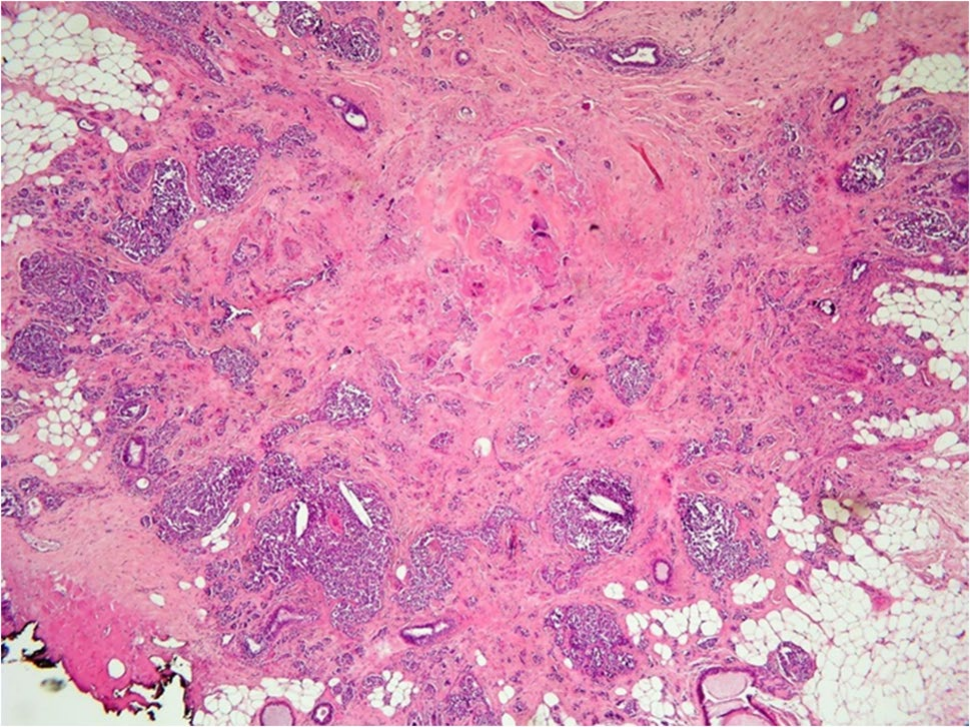
Sheets and nests of round or slightly spindle or plasmacytoid cells with round to oval, regular hyperchromatic nuclei with occasional nucleoli, salt and pepper chromatin in a fibrous tissue. Mitoses were scant (H&E 200X)

**Fig. 4:**
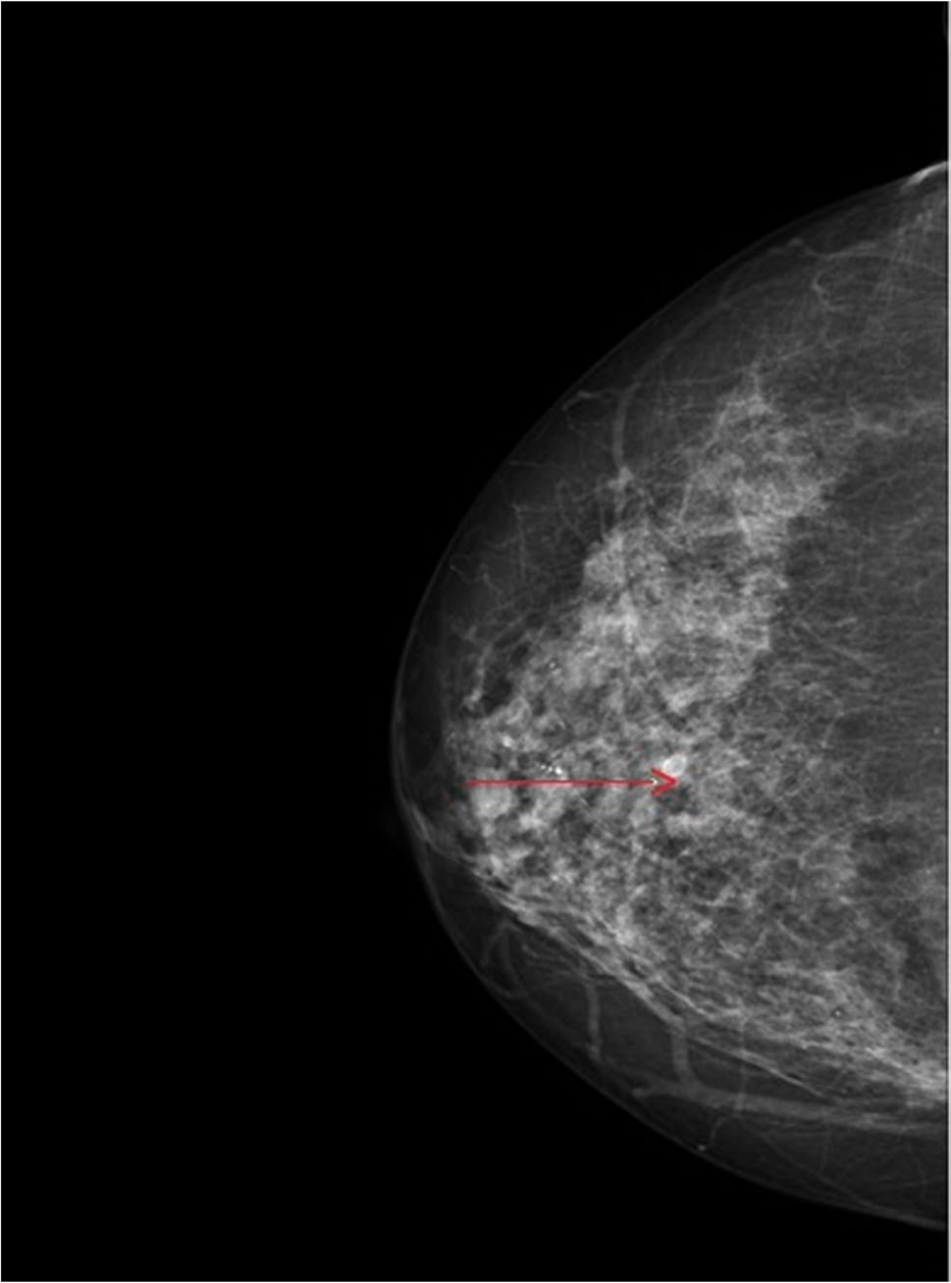
Routine mammography: multiple bilateral calcifications and 4 mm hypo-echoic, homogeneous, roundish nodule at EUQ (RIGHT BREAST), with pale margins, with lobular appearance and not-well defined borders

**Fig. 5:**
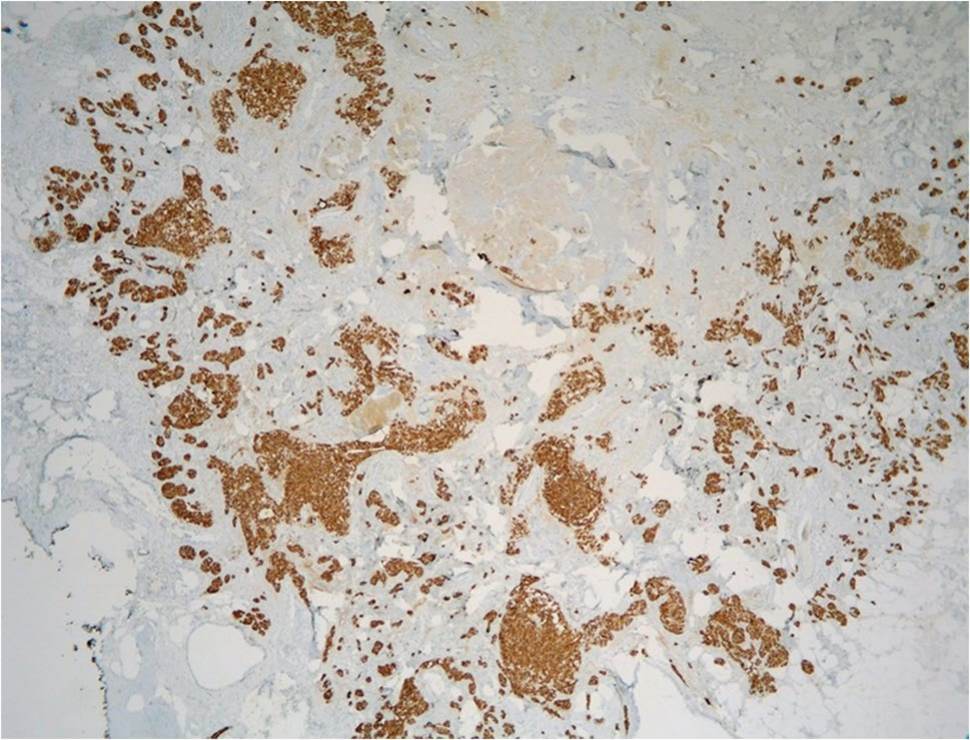
Foci of neoplastic cells with expression of Chromogranin A (AB, 200X)

**Fig. 6:**
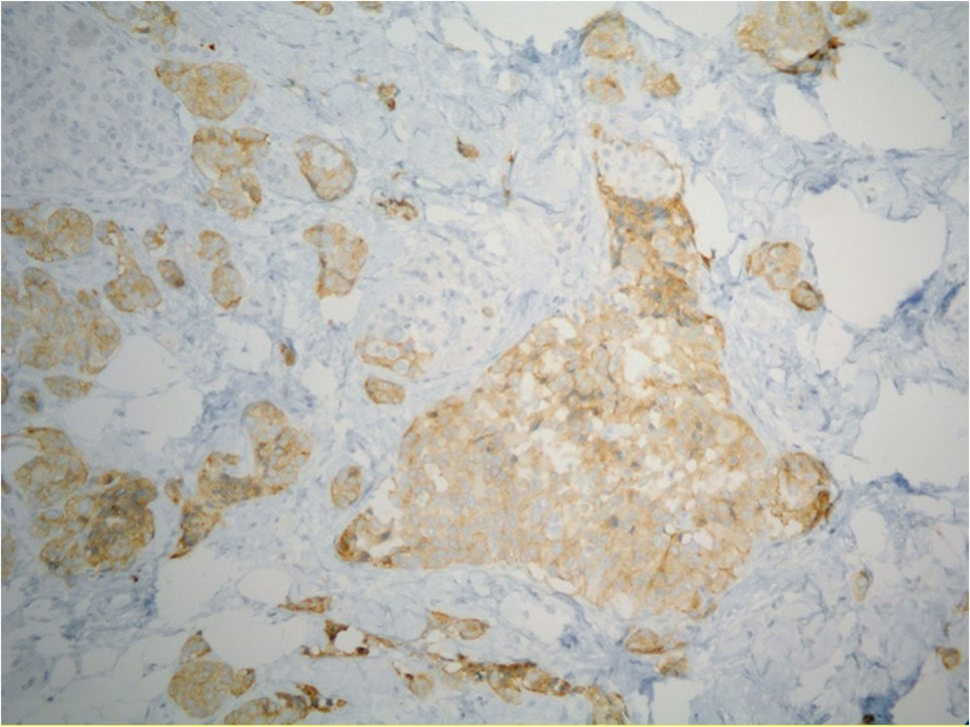
Foci of neoplastic cells with expression of Synaptophysin (AB 400X)

A 54-year-old woman, with no available clinical information, presented with a 7-mm nodule in the upper outer quadrant of the left breast. Histological diagnosis was multiple foci of small cell carcinoma with NE differentiation associated with foci of lobular intraepithelial neoplasm (LIN) 1/2 and a triple-negative immunohistochemical profile (Fig. 1). Eight years after, a new 4 mm nodule was diagnosed as invasive carcinoma with LIN-2 foci (Figs. 2, 3, and 4), showing positivity for CgA and Syn, and a Ki-67 of 5%. Sentinel lymph nodes were negative (Figs. 5 and 6) (Table 1). Surprisingly, it was only after this second surgery that it became known that the patient had been under follow-up and treatment (with somatostatin analogues—SSAs and periodic monitoring of serum calcitonin and CEA levels) for multi-metastatic medullary thyroid carcinoma since 1994. The recognition of this finding, although late, allowed both lesions to be classified as secondary to the original medullary thyroid carcinoma. Given the rarity of this type of neoplasm, careful anatomic-clinical integration is therefore essential to complement the histologic examination (in particular calcitonin immunohistochemical analysis and Congo Red histochemical evaluation have been performed).

#### Case 2

A 67-year-old woman presented with a nodule of the superior-external quadrant of the left breast, which histologically showed solid architecture and small, hyperchromic cells, giving the neoplasm a “blue appearance” (Table 1). The conclusive diagnosis was poorly differentiated carcinoma with small cells and neuroendocrine differentiation with Merkel-like features. Metastasis could not be excluded; in fact, medical history revealed that several years earlier the patient had removed a skin CK20 + nodule, diagnosed as Merkel cell carcinoma. Therefore, the final diagnosis was Merkel cell carcinoma, being metastatic to the breast.

Metastases to the breast affected two patients aged respectively 54 and 67 years. Metastases originated from thyroid medullary carcinoma (case 1, two nodules) and Merkel cell carcinoma of the skin. In both cases, clinical history was of crucial importance for the diagnosis. In case 1, it is of note that lobular in situ neoplasia of classic type was present in the breast parenchyma surrounding the neoplastic nodules. In both cases, ER/PR/Her2 and GATA3 stained negative in the neoplastic cells.

### Primitives

#### Case 3

**Fig. 7:**
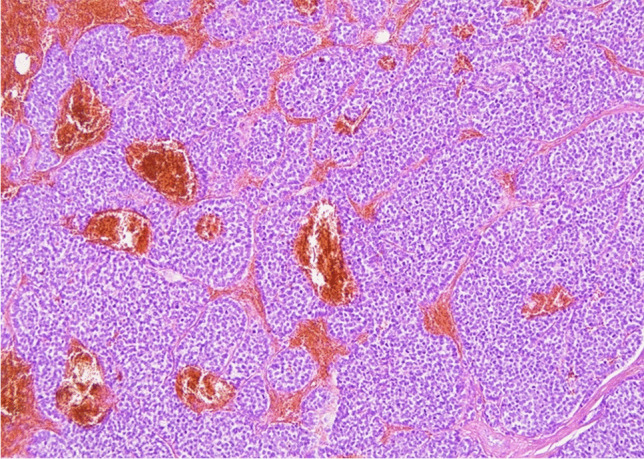
Foci of neoplastic cells with insular pattern (H&E 400X)

**Fig. 8:**
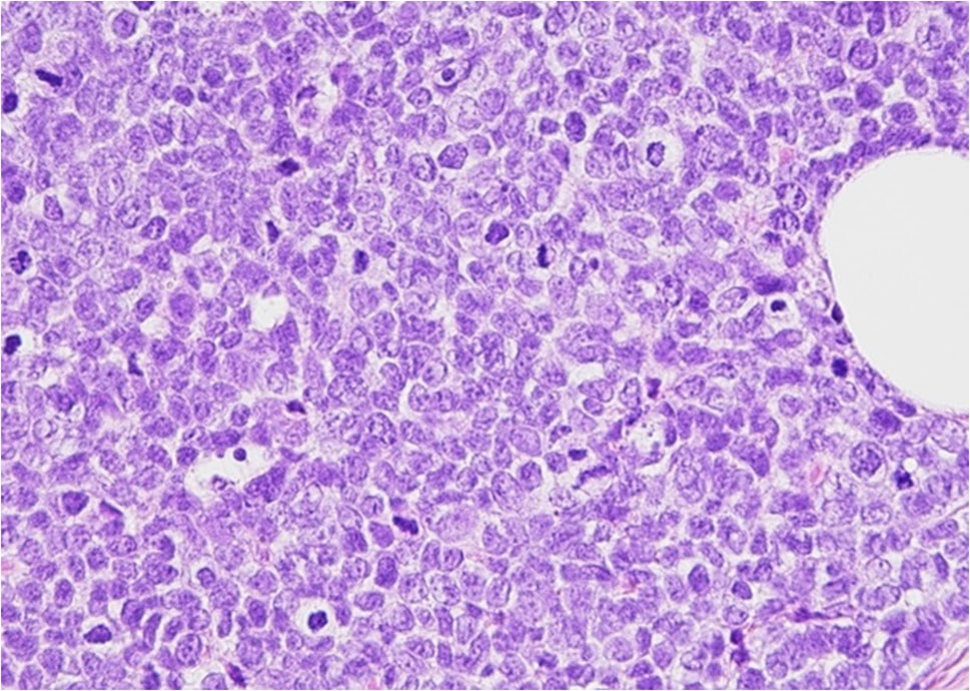
Details about the mitotic activity in the neoplastic foci (H&E 400X)

**Fig. 9:**
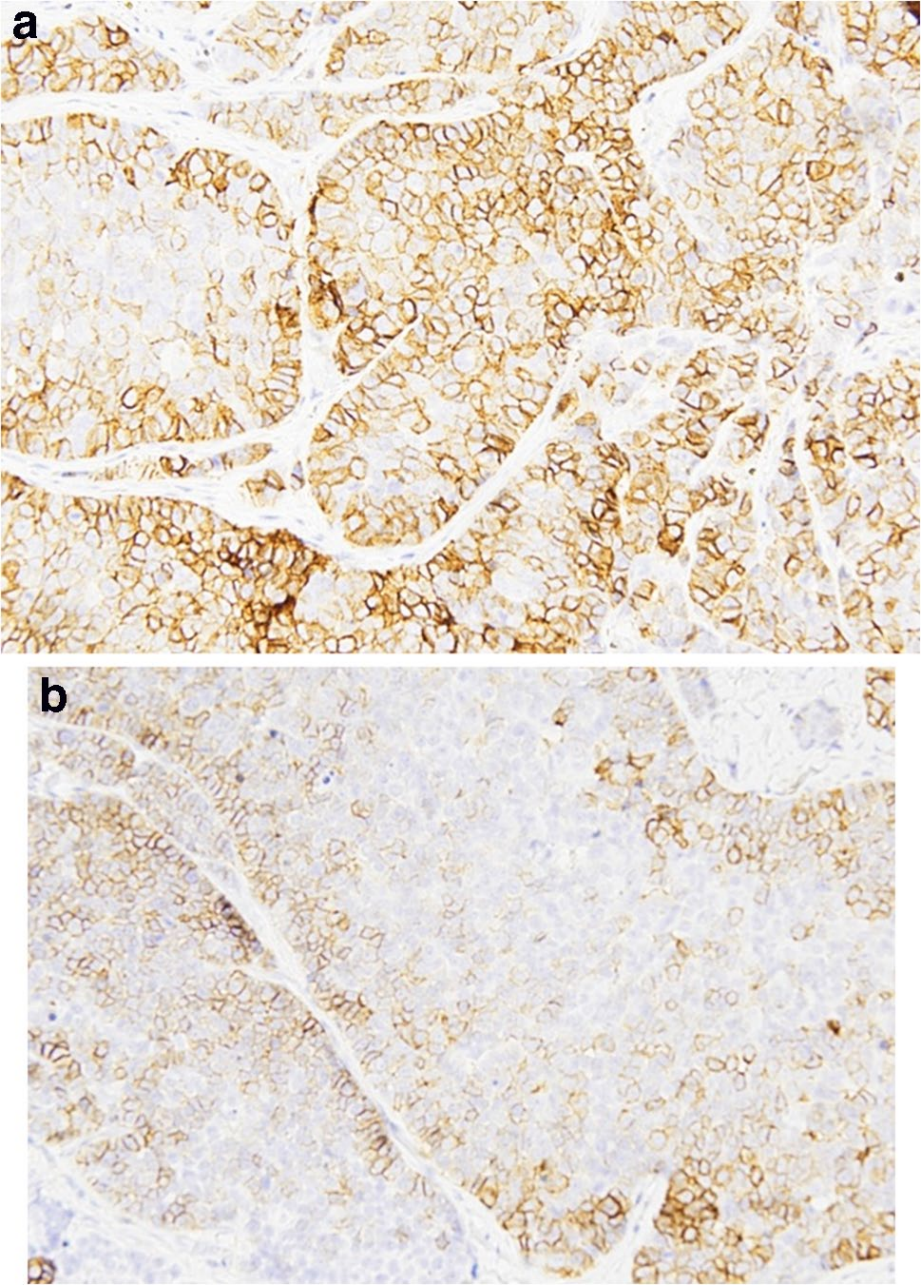
**a** and **b** Details of the expression of SSTR2A with different intensity (strong in **a**, moderate in **b**) (AB 400X)

**Fig. 10:**
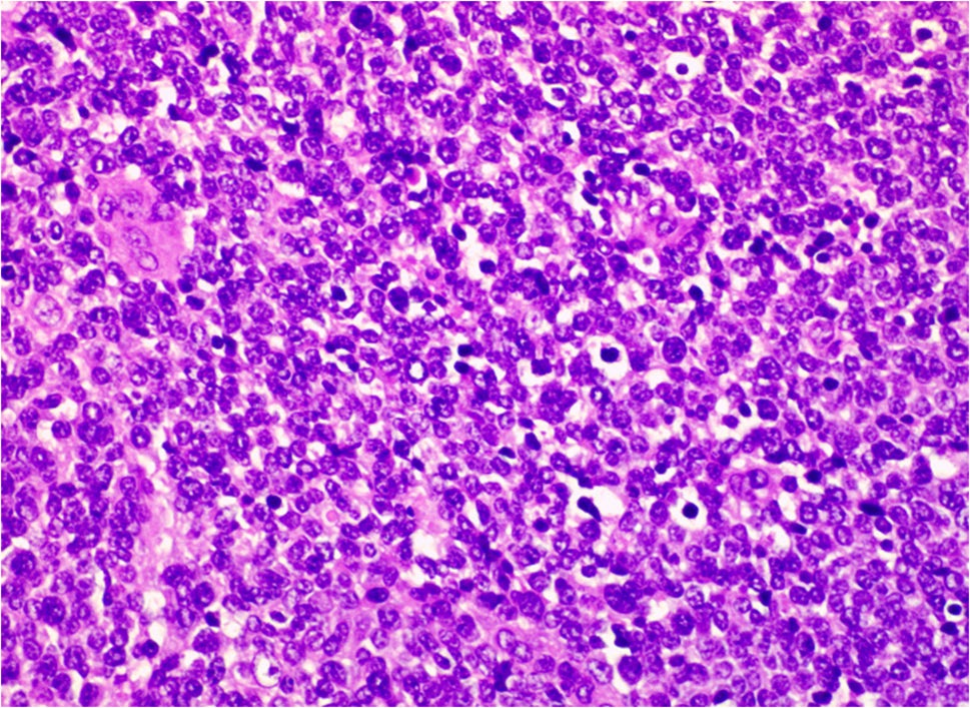
Neoplastic areas with cribriform and papillary architecture (H&E 200X)

A 46-year-old woman presented with a 2.3 cm lump in the superior external quadrant of the left breast. Histologically, the lesion showed an insular architectural pattern consisting of cells with increased nucleus/cytoplasm ratio, pleomorphism, and salt-and-pepper nuclei, with intense mitotic activity (Figs. 7 and 8). The cells were positive for CgA and Syn, SSTR2A, ER (95%), PR (90%), HER2 score 0, and ki-67% of 80%, with a definitive diagnosis of breast poorly differentiated-NEC (Figs. 9 and 10) (Table 1).

#### Case 4

**Fig. 11:**
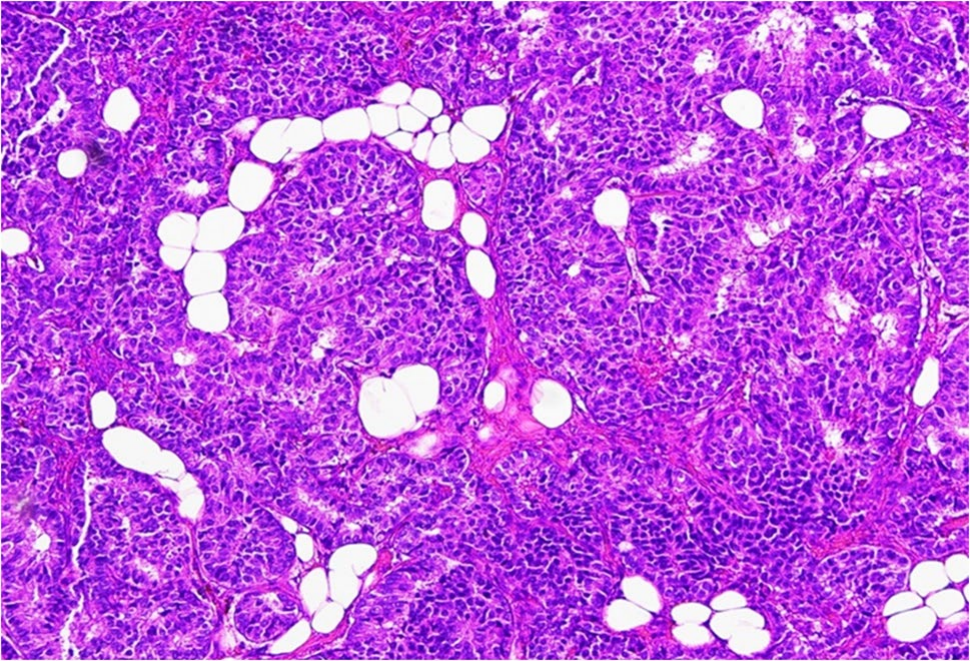
Neoplastic areas with pseudorosette and solid nests (H&E 400X)

A 40-year-old woman showed a 2.2 cm nodule at the inferior-outer quadrant of the left breast. Histologically, a cribriform and papillary architecture were detected, with also solid nests and pseudo-rosettes, associated with a perilesional ductal carcinoma in situ (Table 1), with a final diagnosis of ductal invasive carcinoma G3, with neuroendocrine differentiation (Fig. 11).

#### Case 5

A 36-year-old woman exhibited a 9.5 cm nodule at the superior external quadrant of the left breast. Histologically, it presented with a trabecular, cordonal, and insular pattern, consisting of cells with high nuclear and cytologic pleomorphism, increased nucleus/cytoplasm ratio, numerous mitotic figures, and foci of necrosis. Foci of cribriform and solid ductal carcinoma in situ were observed at the periphery of the lesion (Table 1). In light of morphological and immunohistochemical features, the differential diagnosis for this lesion included a NEN with pulmonary primitivity. However, literature cases [5–7] reported that these immunophenotypic features, if also present in the associated intraductal component, were suggestive of a mammary primary. Thus, the definitive diagnosis was breast poorly differentiated-NEC.

#### Case 6

A 47-year-old woman came to our attention with a 3 cm mass-like area in the inferior outer quadrant of the left breast. Histologic examination showed a lesion with papillary/cribriform ductal invasive NST, and MC type B architecture, consisting of cells characterized by “salt and pepper” chromatin, high nuclear and cytologic pleomorphism, and some mitotic figures (Table 1). The definitive diagnosis was invasive ductal carcinoma G2, with neuroendocrine differentiation.

Four cases presented primary Br-NEN. Patients were all females, aged 36 to 47 years (mean 42).

ER/PR and GATA3 were positive in all cases. HER2 was 2 + in two cases.

## Clinical features

Patients with Br-NEN present clinically in a similar way to patients with IBC-NST. Women of postmenopausal age or elderly are more likely to be affected [9]. No clinical data exist describing how Br-NEN manifests with clinical disorders linked to ectopic hormonal production, including carcinoid syndrome. A considerable proportion of patients diagnosed with NEN present with the disease at stage 2, and they are at a greater risk of developing local lymph node metastases [10–13].

Most cases of Br-NENs in postmenopausal white women in their last seventh decade of life have been reported, 10 years later than the usual types of BC; few cases in males have been described. In contrast to those who have invasive ductal carcinoma not otherwise specified (IDC-NOS) [14, 15].

Radiological features do not substantially differ from those of primary breast cancers, being more similar to those of triple-negative breast carcinomas [16–18]. Somatostatin receptor (SSTR) scintigraphy or positron emission tomography (PET) with somatostatin analogues marked with gallium-68 is useful for predictive purposes if therapy with somatostatin analogues is indicated Care should be taken as SSTR scintigraphy with Indium-111 cannot detect small (< 1 cm) tumours, tumours SSTR negative, or with low affinity for the SSA octreotide [18].

Patients with highly differentiated tumours or advanced cancers may undergo PET-CT with 18-fluorodeossiglucose [19].

## Pathology

### Macroscopic features

At gross examination Br-NENs are nodules with dimension variable from 0.8 to 13.5 cm (mean size of 2.7 cm); they can appear yellowish-coloured, with infiltrative margins or roundish/multilobulated with expansive margins; they can have firm consistency or they can be soft and gelatinous at cut surface, in particular, when if associated with a mucinous component [20, 21].

### Histological features of Br-NECs, Br-NETs, and of invasive carcinomas of no special type with neuroendocrine features

In the 5th WHO classification edition, NEC is defined as a high-grade malignant neoplasm [21], with histological characteristics resembling those of lung SCNEC and LCNEC, respectively. Despite being rare cancer, it exhibits distinctive morphological characteristics: densely packed hyperchromatic cells (cellular streaming) with scant cytoplasm, streaming, and crush artefacts in SCNEC (nuclear moulding is not a prominent feature); large cell with pleomorphic vesicular or hyperchromatic nuclei with irregular membranes and prominent nucleoli in LCNEC.

The precise morphological traits of Br-NETs remain uncertain. The identification of neurosecretory granules and extensive, homogeneous positivity for NE markers is employed to categorise NETs as low- to intermediate-grade invasive tumours with NE differentiation. INSM1 is reported to be a new marker and may aid in the diagnosis of NEN in addition to conventional NE markers [22]. Histologically, fusiform/plasmacytoid/polygonal/spindled cells with abundant eosinophilic and granular/vacuolated cytoplasm to large clear cells, with smooth nuclear borders, inconspicuous nucleoli, and salt and pepper chromatin are arranged in trabeculae and solid, densely packed nests, within a delicate/thin fibrovascular stroma. Ribbons, cords, and rosettes, which are typical characteristics of carcinoid tumours of the lung or NETs in the gastro-entero-pancreatic system, are not always present in Br-NETs. Extracellular mucin deposits or signet ring cells can also be detected. From a clinic-pathological point of view, a diagnosis of Br-NET, although the term tumour, implies an identical treatment to any BC of comparable grade, stage, and hormonal profile, being most Br-NETs hormonal receptors positive.

There is still a lack of clear-cut standardized diagnostic criteria to differentiate real Br-NENs from BCs having some degree of neuroendocrine differentiation. An effective differential diagnosis requires validated and reproducible morphological criteria, and well-defined qualitative and quantitative thresholds for neuroendocrine marker assessment. A previous study demonstrated that breast cancers with neuroendocrine differentiation are frequently misdiagnosed, making their identification challenging. They often lack specific morphological features. In addition, invasive ductal carcinoma and invasive lobular carcinoma (alveolar variant) can mimic some breast cancers with NE differentiation. Furthermore, the impact of NE differentiation in BC on prognosis is unclear, with studies reporting conflicting results [23, 24]. The authors conclude that the correct classification of breast cancers with NE differentiation requires thorough observation of cytologic and structural characteristics and confirmation by IHC. Researchers have also explored the morphological characteristics of breast cancers with NE differentiation [22, 25, 26]. One study compared the characteristics of cancers in which more than 50% of the cells were positive IHC for NE markers with neoplasms of comparable morphology but negative IHC for NE markers. The presence of large, solid, cohesive nests, intermediate nuclear and histological grades, plasmacytoid tumour cells with spindle or columnar shapes, eosinophilic granular cytoplasm, and round nuclei were all identified by the authors as indicators of breast cancer with an NE component [27].

### Diagnostic characteristics of NE-differentiated breast cancers in needle biopsy specimens

Apart from the known challenges in diagnosing NE-differentiated breast cancers by needle biopsy, there is very little literature describing in detail the diagnostic characteristics of NE-differentiated breast cancers in needle biopsy specimens. NETs are distinguished by loosely coherent sheets of orderly cells with plasmacytoid, eccentric granular cytoplasm, spherical nuclei with spotted “salt and pepper” chromatin, and discrete nucleoli, among other cytologic characteristics. The NEC resembles NECs developing at other places as well as the SCNEC of the lung. Further research is therefore required to accurately characterize and categorize breast cancers with NE differentiation [28].

### Pure and mixed forms

Finally, in the breast parenchyma generally, the diagnosis of pure Br-NENs can be applied in a small subset of cases, whereas mixed forms, in which NEN component co-exists with an NST or special type BC, are more common. However, although mixed neoplasms (mixed neuroendocrine/non-neuroendocrine neoplasms, MiNENs) are considered an integral part of NENs in the digestive tract and other organs, the 2019 WHO classification of breast tumours does not consider this entity as a NEN.

### Immunohistochemistry and molecular subtype

Immunohistochemical analysis of NE biomarkers represents the gold standard in the diagnosis of NENs. Together with the most sensitive and specific markers, namely CgA and Syn, recently, a novel biomarker, INSM1, has been proposed as an accurate indicator of NE differentiation to support NEN diagnosis, in particular, in poorly differentiated neoplasms [29]. However, these immunomarkers are not routinely assessed, whilst they are reserved only when a well-trained / expert pathologist identify or suspect a NE morphology in H&E-stained routinary slides.

We must remember that none of the above-mentioned NE markers proved to be useful in clearly distinguishing pure Br-NENs from other breast carcinomas with NE differentiation [30]. As a result, the diagnostic criteria, the proposed cut-off for NE immunomarkers, and the terminology of Br-NEN have varied in recent studies [23] creating limitations for the correlation of data. Well-differentiated NETs and poorly differentiated NECs are more likely to show diffuse NE marker positivity than IBC-NEDs.

ER/PR/AR markers show positivity in most well-differentiated NETs and in greater than 50% of poorly differentiated NECs *(most NETs fall into the luminal B molecular subtype and molecularly cluster together with mucinous A-B tumors)*. Poorly differentiated NECs of the breast often show expression of TTF1 (generally well known as a lineage marker of lung origin) and up to 45% of them also show expression of AR, often co-expressed with GCDFP15. Negativity for basal markers (CK5/6, CK14, p63) as well as the EGFR protein has been observed. CDX2 consistently shows negativity in primary breast NETs, so it could be useful to differentiate it from a gastro-intestinal primary. Other markers such as Calcitonin, CEA, PAX8 (variable and weak) [31], and the Congo red for amyloid, together with a triple-negative phenotype (ER-, PR-, HER2 score 0) can aid in diagnosing a breast metastasis from a medullary thyroid cancer,

SSTR expression in breast NENs, similarly to extra-mammary NENs, is a well long-known phenomenon, potentially allowing SSTR-based tumour imaging (octreoscan or 68 Ga-DOTATOC PET/CT) and SSTR-targeted tumour treatment (octreotide or lanreotide) [30]. Specifically, SSTR1-5 are G-protein-coupled plasma membrane receptors with 7 trans membrane regions; among them, SSTR 2A is a subtype overexpressed in the majority of NE tumours and also most commonly expressed in BC (6, being able to mediate the antiproliferative effect of molecular-targeted therapy with SSA in the strongest manner [32].

However, the SSTR 2A positivity rate in BC-NENs has only been analysed in two studies [23, 33]. These recently published retrospective analyses of selected NENs reported a SSTR 2A positivity rate of 71% and 50%, respectively [23, 33]. SSTR 2A, when overexpressed, can be a good candidate for the targeted therapy with SSA such as octreotide or lanreotide (antisecretory treatment and antiproliferative activity in functional NENs). In extra-mammary NENs, this therapy is mainly being considered in well-differentiated NETs (G1/2, Ki-67 < 10%) [34, 35]. Current recommendations for Br-NENs therapy rely on general guidelines for breast cancer. Only SCNEC has specific therapeutical recommendations (i.e., platinum/etoposide-based chemotherapy similar to small cell lung cancer). SSA therapy has been evaluated in BC-NST and seemed to show response rates of up to 40% in a metastatic setting in phase I – II trials [36]. However, a phase III study comparing endocrine therapy with or without octreotide in primary ER + BC did not show a real clinical benefit of SSA treatment in this setting [37]. Thus, outside of the context of the exceedingly rare SCC of the breast, NE differentiation in breast neoplasms seems to be not regarded to have therapeutic significance.

### Molecular features

From a molecular point of view, Br-NENs have been considered a distinct subtype of luminal (generally) breast carcinoma, occurring predominantly in post-menopausal women. They are ER (100%), PR (89%), GATA3 (98%), FOXA1 (96%), and CK8/18 (98%) positive. There was an almost equal distribution of luminal A (52%) and B (48%), with a low rate of PIK3CA and p53 mutations, lack of concurrent 1q gains/16q deletions, reported trisomy of chromosomes 7 and 12 and FGFR mutations, high frequency of GATA3 mutations and an aggressive clinical behaviour, representing a histologically and genomically entity related to MC, distinct from ER + /HER2- IBC-NST. There was no evidence of gene amplification in cMET, EGFR, or TOP2A. Targeted sequencing of 47 genes discovered variations in the TP53, PIK3CA, ERBB4, and APC genes. Gene expression data (including the somatostatin receptor gene family—SSTR1/2/3/4/5) were available for 5 patients, for which 3 out of 5 patients showed overexpression of at least one SSTR gene.

## Differential diagnosis

Breast NENs have a wide range of potential diagnoses, including both benign and malignant conditions. Primary breast NEN can potentially be diagnosed as metastasis from other organs. Similarly, metastatic neuroendocrine neoplasm of extra-mammary location in the breast setting is the most significant differential diagnosis that must be ruled out [23, 38–40]. Metastatic NENs from an extramammary site represent 1 to 2% of secondaries to the breast. In one review, 25 up to 44% (8 of 18) of metastatic NENs to the breast were misdiagnosed as primary breast cancers [40]. The distinction of primary from metastatic NEN is critical to avoid misdiagnosis and unnecessary surgical and medical therapy in the latter.

Primary Br-NEN are morphologically similar to NENs arising in other organs. Features indicating a primary breast origin are the positivity of site-specific lineage markers indicating breast origin as ER/PR/AR, GATA3, mammaglobin, GCDFP15; axillary N + ; lack of a history of an extramammary primary NEN; nuclear atypia or pleomorphism; the presence of a surrounding in situ component. However, in rare cases, similarly to Case 1 of our series, the detection of an in situ component seems to contradict this concept: specifically, in our case, an in situ lobular carcinoma was present around the metastatic thyroid medullary carcinoma. On this wave, careful clinical history and whole-body radiological imaging are of outmost important to correctly address the diagnosis. In doubtful cases, organ-specific markers can be of help [38–42].

Regarding the molecular subtype, most breast NENs are hormone receptor-positive and human epidermal growth factor receptor 2 (HER-2)-negative, presenting a luminal-like phenotype, so that ER might help to distinguish BR-NEN from possible metastasis. On the other hand, we have to consider that hormonal receptors may also be positive in metastatic neoplasms. Consequently, the diagnosis of BR-NENs is made on histology and IHC staining of neuroendocrine markers (Syn, CgA, INSM1), which should be always performed in the suspicion of a neuroendocrine differentiation, and supported by clinical and imaging data [38–40].

In routinary practice, breast carcinomas with neuroendocrine features are frequently underdiagnosed. This rate of misinterpretation is not surprising given the variable neuroendocrine features of primary breast NENs and the absence of standard testing for neuroendocrine markers. Misdiagnosis of neuroendocrine features in IDC-NOS or in lobular invasive carcinoma is unlikely to be significant, given the fact that there is no agreement on the clinical and prognostic implications of neuroendocrine differentiation [38–40].

Endocrine ductal carcinoma in situ (E-DCIS) and SPC share usual ductal hyperplasia (UDH) as a differential diagnosis. Both solid papillary carcinoma and E-DCIS have a solid growth pattern and cells that arrange themselves in a pattern like UDH. Because neuroendocrine marker positivity is not seen in benign breast lesions, it validates a diagnosis of E-DCIS or solid papillary cancer. UDH will also be ruled out if cytokeratin 5/6 is negative and ER is diffusely positive (clonal-type). Solid papillary carcinomas, unlike UDH, are negative for myoepithelial markers (smooth muscle myosin, actin, and p63) [23, 38–41].

### Therapy

Some claimed that treating Br-NENs, particularly NETs, in the same manner as NENs originating in other organs, requires careful consideration because current Br-NETs lack a clear-cut morphology and rely on IHC for CgA and Syn, which can occasionally be expressed in other non-NENs as well. The authors also noted that breast NETs and non-NEN BCs exhibit similar clinical behaviours and therapeutic responses and that BrNETs and luminal type A breast cancer exhibit more similarities than NETs derived from other organs, according to molecular and genetic analyses.

NENs behave similarly to other invasive BCs so that the treatment plan is also based on prognostic and predictive factors, such as the TNM stage, ER and PgR receptor status, HER2 status, genotypes and nuclear grade, Ki67 index, age, menopausal status, and general health conditions, such as those that apply to another breast invasive tumours. One of the first distinct in treatment is between localized and metastatic disease. A minority of patients have a localized disease with no randomized trials to guide the management mostly due to the rarity. The management and therapeutic approach are mostly based on retrospective studies and/or case reports. However, surgery represents the first option for localized disease, similar to ductal and lobular cancers. Furthermore, adjuvant chemotherapy and chemoradiation are both used after surgery mostly depending on the tumour size, lymph nodal involvement, and metastases. In the next paragraphs, we briefly discuss some of these aspects in the management of these lesions.

According to the IBC-NST, both adjuvant and neoadjuvant chemotherapy are equally beneficial in reducing the risk of a distant recurrence and the mortality rate from brain cancer [40] In recent years, the practice of treating cases by escalation or de-escalation, in which postoperative care can be tailored based on the efficacy and responsiveness to neoadjuvant therapy, has also come to be accepted [42–45]. In cases of metastatic disease, palliative systemic chemotherapy is the main treatment.

### Surgery

According to the St Gallen guidelines, and regardless of the histotypes, women presenting with screen-detected or other early breast cancers are potential candidates for breast-conserving surgery [46] Nevertheless, several patients prefer a mastectomy including contralateral mastectomy mostly associated with the fears of recurrence, improvements in reconstruction techniques, more widespread use of MRI imaging during the diagnostic evaluation, genetic testing and lack of adequate physician/patient communication [47] Specifically, patients with resectable Br-NENs are advised to undergo surgery. To choose the best surgical strategy, it is crucial to distinguish between primary NENs and metastatic NENs from other organs [48–50].

### Chemoradiotherapy

Adjuvant systemic therapy should be used based on each patient’s clinicopathological features and risk of recurrence. Data from literature are not sufficient to compare the benefit of adjuvant platinum-based versus taxane-based and/or anthracycline regimens [51]. The majority of studies, showing the long-term survival benefit of etoposide plus cisplatin or carboplatin have been derived from small-cell lung cancer studies [52]. Also, the number of cycles, six or four, is not well assessed and it is mostly based on patient tolerance [52]. Although the efficacy of adjuvant chemotherapy in patients with Br-NENs has not been reported in specific clinical trial reports, patients with high-risk disease should be treated with adjuvant or neoadjuvant chemotherapy on an individual basis. The key considerations when selecting whether to start adjuvant or neoadjuvant chemotherapy for patients with IBC-NSTs are tumour size, nodal status, nuclear grade, age, tumour subtype determined by IHC for ER, PgR, and HER2, and the Ki67 index. Tumour size and nodal status are also important predictors of recurrence in patients with Br-NENs [53].

Based on findings from GI NETs, breast NETs may be less sensitive to chemotherapy (CHT) than IBC-NSTs [54–58]. For the metastatic patients, the first line of chemotherapy has been derived from small-cell lung cancer, with etoposide plus platinum as the first standard approach [59, 60]. More clinical trials are needed to determine whether the anthracycline plus taxane combination is a viable therapeutic option for people with high-risk Br-NENs.

Escalation therapy or response-guided treatment is being developed primarily in TN breast cancer. Adjuvant capecitabine treatment for 6 months enhanced disease-free survival in patients with remaining triple-negative breast cancer following conventional neoadjuvant chemotherapy [61].

Radiotherapy (RT) for the chest wall and regional lymph nodes should be given in the same way that it is for IBC-NSTs [45, 62, 63]. There have been no reports of clinical trials of radiation following surgery for Br-NEN patients. One case-controlled study found that RT improved survival in patients with NEC of the breast as defined by the WHO 2003 classification [45], whereas another population-based study found that adjuvant radiotherapy did not improve survival in patients with primary SCNEC of the breast [55].

In conclusion, basically, today, neuroendocrine neoplasms, except small cell type, do receive similar systemic treatments as stage and biomarkers matched NST carcinoma with or without neuroendocrine differentiation.

## Hormonal and molecular therapy

According to case studies, Br-NENs are usually HR-positive, making hormone therapy a viable treatment option [11, 64, 65]. Breast NETs treated with hormonal therapy (HT) and RT had longer overall survival (OS) and disease-free survival (DFS) than those who did not receive, while patients who received CHT had lower OS and DFS than those who did not.

Adjuvant hormone therapy is recommended for 5–10 years for IBC and NST [65], and is also indicated for HR-positive Br-NEN. Combining an aromatase inhibitor or fulvestrant with a CDK4/6 inhibitor resulted in a significant improvement in progression-free survival and overall survival compared to hormonal therapy alone in important clinical studies in patients with HR-positive IBC-NSTs [66–72].

Recent molecular studies have found PIK3CA mutations in 7–33% of Br-NENs, which is lower than the prevalence reported in HR-positive HER2-negative IBC-NSTs [73–76]. A conventional therapy strategy for HR-positive, HER2-negative IBC-NSTs is to target the PI3K/AKT/mTOR pathway using a PI3K inhibitor (e.g., alpelisib) and a mTOR inhibitor (e.g., everolimus) in different studies [77–80] Interestingly, oncogenic, or possibly oncogenic PI3K pathway mutations were more prevalent in NETs than in NECs (50% vs. 18.2%). Targeting the PI3K/AKT/mTOR pathway may, therefore, be a realistic and promising method for treating HR-positive HER2-negative Br-NENs, particularly NETs.

Anti-HER2 therapy is appropriate for HER2-positive Br-NENs. Although there is minimal evidence that HER2 status predicts NENs, there is a case report in the literature of the success of trastuzumab treatment in a patient with NEC and HER2 amplification of the breast [81]. Another instance with well-differentiated NET of the breast with HR-positive HER2-positive status was treated with surgery, adjuvant CHT, trastuzumab, and HT, resulting in disease-free status after 9 years of follow-up [82]. Several other anti-HER2 agents and an antibody–drug conjugate have been established as standard treatments for HER2-positive IBC-NSCLC in both the adjuvant and metastatic settings [83–91].

Recent clinical studies have shown that novel antibody–drug conjugates aimed against HER2 offer considerable therapeutic advantages in the treatment of this kind of malignancy [92, 93]. There are presently no clinical trials for the use of drug conjugates for neuroendocrine breast carcinomas with HER2 low immunophenotype. An interesting evidence from our cohort is that around 33% of our patients in our relatively small case series had a HER2 low immunophenotype.

Somatostatin analogues are an essential therapeutic option in the diagnosis and treatment of gastro-entero-pancreatic NETs. IHC has also revealed that breast NETs are positive for somatostatin receptor types 2, 2A, 2B, 3, and 5 [94]. While it can be a successful therapy option, somatostatin analogues have not yet shown clinical efficacy in the treatment of breast NETs [95]. SSTR2A can be assessed by immunohistochemistry, and it is given a score according to the following characteristics: Score 1 included pure cytoplasmic reactivity, either moderate/strong or weak; Score 2 was assigned when there was a membranous pattern of staining in less than 50% of tumour cells, either in scattered cells with complete membrane outlining or in most tumour cells with partial membrane staining; Score 3 was assigned when there was a membranous, usually intense, staining in more than 50% of tumour cells [96].

PRRT using radiolabelled somatostatin analogues for somatostatin receptor-targeted PET-CT has proven efficacy for NENs expressing somatostatin receptors and is predicted to be a promising therapeutic method as shown by two case reports [97, 98].

Immune checkpoint inhibitors are being developed for breast cancer and have already established the standard of care for TNBC with PD-L1. Because single medications' efficacy has been insufficient, combination therapy with chemotherapy or other targeted therapies is now being researched [93–96, 99–102]. For Br-NENs, there is no clinical data on immune checkpoint inhibitors.

Phase 1/2 trials were carried out to assess the role of immune checkpoint inhibitors in NENs, and the results revealed promising efficacy and controllable toxicity [97, 103]. To explore the use of immune checkpoint inhibitors, it is reasonable to assess the PD-L1 status of HR-negative HER2-negative breast NEC.

## Prognosis

Most studies published in the literature looked at NE breast neoplasms as a whole, without classification into various subtypes (as recommended by the WHO). As a result, data on the predictive significance of NE features in breast cancers is inadequate to be definitive. The question whether neuroendocrine differentiation affects the prognosis of BC patients remains a very much debated issue, with contrasting clinical results. The majority of published large series demonstrated an impaired prognosis for NENs and that neuroendocrine differentiation is an independent adverse prognostic factor for OS and DSS in BC, with a higher rate of local and distant recurrence although some smaller studies reported similar or even better outcomes for NEN compared to BC-NST patients (21) SCNEC had the worst outcome of any NE breast cancer [104–109]. The better prognosis of NETs in the breast compared to SCNECs is due in part to their lower histologic Nottingham grade. Although well-differentiated NETs had considerably better long-term results than non-NE tumours in other anatomic regions, there was no data to support comparable findings in the breast until today.

Histologic categorization and Nottingham staging are predictive factors in NEN, as they are in IBC. The Ki67 proliferation index, while beneficial in other NE tumours, is not predictive in NE breast cancers. The prognosis for IBC-NST with NE differentiation is less known. One explanation for this is that this subgroup is extremely diverse, with examples of varied NE marker expression and NE morphologic differentiation. Numerous studies that have indicated poorer outcomes in NE breast cancers (defined by NE marker expression) may have included numerous instances of IBC-NST with NE differentiation. [106].

It is debatable if the degree of expression of NE markers has any prognostic relevance. The presence of focused (as opposed to diffuse) NE markers, notably chromogranin (CG), was found to be a predictor of poor outcome [107]. Other studies that used a higher NE marker threshold or Tissue Microarray (TMA) analysis found no difference in survival, although their case selection may have underestimated instances with extremely low expression. Surprisingly, Cga/Syn ^pos^ breast tumours that also expressed other NE markers, such as DCLK1 and INSM1, had a better prognosis. Overall, a higher amount of NE differentiation appears to be associated with a better prognosis. Individual markers, but not other NE markers, were revealed to represent an independent negative prognostic predictor [108].

Because more thorough research is needed, these findings were preliminary yet intriguing. At this stage, it may be appropriate to advise that when diagnosing NEN, it is wise to distinguish between NET and NEC and to record the amount of expression of NE markers; the use of other markers (e.g., INSM1) may further stratify patients into various prognosis groups [109, 110]. Future studies are more likely to show more solid prognostic information in Br-NENs with uniformity of diagnostic criteria and categorization [95, 96]. Up to now, since neuroendocrine differentiation has been shown to be associated with impaired outcomes in several retrospective trials, further studies are needed to identify the most appropriate treatment strategy for this BC subtype [111, 112].

## Conclusions

Br-NENs are a special type of BCs with NE cytomorphological features and positivity for NE markers, mostly clustering with the luminal molecular profile. NENs are described in one specific chapter in the 2019 WHO classification of breast tumours. Br-NENs can occur in pure form or mixed forms. The real incidence of Br-NENs is difficult to define since NE markers are not routinely used in breast cancer diagnostic paths and due to the different data collection procedures. Thus, NENs of the breast are mis-diagnosed or under-recognized accounting for < 1% of BCs. According to the recent 2022 WHO classification of neuroendocrine neoplasms, Br-NENs can be distinguished into two families: well-differentiated forms (WD-NET) and poorly differentiated forms (NEC) according to morphology. We must keep in mind to be always careful to rule out metastatic nature from other origins (in particular excluding metastatic NEN from an extramammary site). The distinction of primary from metastatic NEN is critical to avoid misdiagnosis and unnecessary surgical and medical therapy. No consensus has been reached on the prognosis for Br-NENs and the efficacy of targeted therapies should be furtherly studied.

## Data Availability

There is a word file including the details of our cases.

## Abbreviations

BC: Breast carcinoma
Br-NENs: Breast neuroendocrine neoplasms
CHT: Chemotherapy
DFS: Disease-free survival
E-DCIS: Endocrine ductal carcinoma in situ
GI/NETs: Gastro-intestinal neuroendocrine tumours
HER-2: Human epidermal growth factor receptor 2
HT: Hormonal therapy
IBCs-NST: Invasive breast cancers of no special type
IDC-NOS: Invasive ductal carcinoma- no specific type
IHC: Immunohistochemistry
LCNEC: Large cell neuroendocrine carcinoma
LIN: Lobular intraepithelial neoplasm
MC: Mucinous carcinoma
MIBG: Meta-iodo-benzyl-guanidine
MiNEN: Mixed neuroendocrine/non-neuroendocrine neoplasm
NE: Neuroendocrine
NEN: Neuroendocrine neoplasm
NEC: Neuroendocrine carcinoma
OS: Overall survival
PRRT: Radionuclide therapy with peptide hormones
PET: Positron emission tomography
RT: Radiotherapy
SCNEC: Small cell neuroendocrine carcinoma
SPC: Solid papillary carcinoma
SSA: Somatostatin analogues
SSTR: Somatostatin receptor
TC: Computerized tomography
TMA: Tissue microarray
TN: Triple negative
UDH: Usual ductal hyperplasia
WD-NETs: Well differentiated – neuroendocrine tumours
